# Domains and determinants of retirement timing: A systematic review of longitudinal studies

**DOI:** 10.1186/s12889-018-5983-7

**Published:** 2018-08-31

**Authors:** Micky Scharn, Ranu Sewdas, Cécile R. L. Boot, Martijn Huisman, Maarten Lindeboom, Allard J. van der Beek

**Affiliations:** 1 Department of Public and Occupational Health, Amsterdam UMC, VU University Amsterdam, Amsterdam Public Health Research Institute, Van der Boechorststraat 7, NL-1081 BT Amsterdam, The Netherlands; 2 Department of Epidemiology and Biostatistics, Amsterdam UMC, VU University Amsterdam, Amsterdam Public Health Research Institute, De Boelelaan 1089a, NL-1081 HV Amsterdam, The Netherlands; 3 Department of Sociology, VU University Amsterdam, De Boelelaan 1089a, 1081 HV Amsterdam, The Netherlands; 4 Department of Economics and Development Economics, VU University Amsterdam, De Boelelaan 1105, 1081 HV Amsterdam, The Netherlands

**Keywords:** Cohort studies, Older workers, Pension, Economics, Occupational health

## Abstract

**Background:**

To date, determinants of retirement timing have been studied separately within various disciplines, such as occupational health and economics. This narrative literature review explores the determinants of retirement timing in countries, and relevant domains among older workers from both an economic and occupational health perspective.

**Methods:**

A literature search was conducted using 11 databases. Longitudinal studies on determinants of retirement timing were included. Study inclusion criteria were as follows: full-text article written in English or Dutch, conducted in humans, main outcome was time until retirement (i.e. retirement date or retirement age), and longitudinal design. Next, the included articles were screened for hypotheses on retirement timing and these articles with hypotheses were subjected to a quality assessment. Determinants for retirement timing were classified into multiple domains by three researchers.

**Results:**

The literature search identified 20 articles. The determinants of retirement timing were classified into eight domains: demographic factors, health factors, social factors, social participation, work characteristics, financial factors, retirement preferences, and macro effects. In total, we identified 49 determinants, ranging from one (social, and retirement preferences) to 21 determinants (work characteristics) per domain.

**Conclusions:**

The findings suggest that there is a wide range of determinants that influence retirement timing in modern industrialized countries and that these determinants differ between countries. We recommend that researchers include determinants from various domains when studying retirement timing, while taking into account a country’s context.

**Electronic supplementary material:**

The online version of this article (10.1186/s12889-018-5983-7) contains supplementary material, which is available to authorized users.

## Background

In many modern industrialized countries, the population is ageing rapidly and individual life expectancy is increasing. Similarly, the ratio of the population aged 65 years and above to the population aged 20-64 years (i.e. old age dependency ratio) is increasing. Among the Dutch population, this ratio will double from 27.2% in 2012 to 52.5% in 2050 [1]. These developments have caused pressure on social security systems. For this reason, governments have been implementing policy changes to prevent an early exit from the workforce and increase the retirement age. In the coming years, the statutory retirement age in half of the OECD countries will be 65 years, and in 14 countries it will be between 67 and 69 years [2]. In the Netherlands, for example, the statutory retirement age is gradually being increased from 65 years in 2012 to 67 years in 2021[3].

The retirement trends of older workers in modern industrialized countries have changed over the past decades. During the second half of the twentieth century, there was a strong trend towards early retirement. This early retirement trend in the year 1995 was most pronounced in the Netherlands, Belgium and France with average retirement ages below 60 years [4]. Moreover this trend was also evident in the United States, since the average retirement age for men decreased from 70 years in 1940 to 63 years in the early 1980s [5]. This trend ended and even reversed in the mid-1990s, and 2000s [6]. Nevertheless, many workers in most OECD countries still leave the workforce before the official retirement age of 65 years. To illustrate, in France, United Kingdom, Germany and Denmark, workers retired at ages 59.4, 64.1, 62.7 and 63.0 respectively from 2009-2014 [2].

Although older workers are stimulated by recent policy changes to retain in the workforce until higher age, it remains unclear what the determinants are for retirement timing among older workers. Retirement timing has been shown to be a complicated process of labour force exit [7, 8]. This process of labour force exit includes all the determinants that people take into account when deciding when they want to retire. The determinants of retirement timing have been studied within various disciplines, such as occupational health and economics. These studies have focused mainly on determinants relevant to their own discipline. Examples of determinants in the occupational health literature have been mostly related to the health or work domains (e.g. self-perceived health and work ability), whereas determinants investigated in economics have been mainly in the financial domain or related to effects of policy reforms [7, 9–12]. Furthermore, several frameworks have been developed to capture the complexity of the decision-making process about retirement, such as the leben in der Arbeit (lidA) framework and the research framework of the Study on Transitions in Employment, Ability and Motivation (STREAM) [13–15]. To date, no systematic review has been performed on the literature available in both disciplines regarding the determinants of retirement timing (i.e. time until retirement and/or the age at which people retire), and the main domains of the determinants. Therefore, the aim of this narrative literature review was to present an overview of determinants used in retirement timing research from an economic and occupational perspective, and to cluster these determinants into domains. Additionally, we aimed to identify gaps and recommendations for further research on retirement timing.

## Methods

A literature search was conducted to identify relevant articles with determinants on retirement timing. Components from the PRISMA statement [16] were used in reporting this systematic review.

### Search strategy

The following databases from the disciplines of occupational health and economics were used: Web of Science, Embase, PsychINFO, CINAHL, Pubmed, IBSS, ABI/Inform Global, Business Source Elite, ECON Papers, PICARTA, and Grey Literature in the Netherlands. The search was carried out on July 22, 2015. The following search terms were included: retirement timing, early retirement, retirement anticipation, retirement expectation, retirement preparedness, transition to retirement or retirement application. The complete list of Web of Science search terms is presented in Additional file 1. The search strategies for the other databases were based on the same search terms. After removing duplicates, all titles and abstracts were screened. Based on the selection criteria described below, the full text was screened and afterwards a reference check was performed on the included articles. Since the research team was mainly embedded in occupational health, a consultation meeting was held with an expert in economics to discuss the included articles and to consider missing articles.

### Selection criteria

All titles and abstracts were screened by two independent reviewers (MS & RS). If consensus between the two reviewers could not be reached, the abstract or full text was screened by a third reviewer (CB) and this reviewer made the final decision. Full-text articles were retrieved for further assessment if the study abstract met the following inclusion criteria: a full-text article written in English or Dutch, was conducted among humans, the main outcome was time until retirement, and a longitudinal design was used. Full-text articles were also retrieved for further assessment if there was no abstract available or no consensus between the two researchers. Next, the included articles were screened for hypotheses regarding retirement timing.

Studies were excluded if exit routes from work were not further defined or if the main outcome was disability pension. Moreover, cross-sectional studies were excluded in the present study, because time was not taken into account. In the final step, studies that did not test hypotheses related to determinants of retirement timing were excluded, but these were assessed in the sensitivity analysis.

### Quality assessment

The results of some studies are more likely to be biased than others due to differences in methodological quality between studies. Therefore, the quality of a study must be taken into account. Two reviewers (MS & RS) independently assessed the quality of the included studies. The quality of the six articles used in the sensitivity analysis was not assessed. The standardized checklist was based on the checklists of Hayden [17]. Table 1 shows the standardized checklist for the methodology quality. Each item was scored as positive (+) or negative (-). Negative is seen as potential bias. If the paper provided insufficient information on the specific item, the item was scored with a question mark. If an item was not applicable, it was scored as NA. Disagreement between the reviewers was identified and solved during a consensus meeting. The total quality score consisted of the items rated positive divided by the total number of applicable items. Based on the total score, studies were either seen as high (>50%) or low quality (≤50%). High quality studies were assessed as having a low risk of bias, while low quality studies are assessed as having a high risk of bias. This is in line with other studies [18, 19].

**Table 1:** Checklist of methodological quality

Study objective	
1	Positive if a clearly stated objective is described
Study population	
2	Positive if the main features of the study population are clearly described
3	Positive if the inclusion and exclusion criteria are described
Outcome	
4	Positive if a clear definition of retirement (timing) is given
5	Positive if outcome source is register-based
Determinants	
6	Positive if adjusted for other confounders/determinants from different scientific fields
7	Positive if age (if possible), gender (if possible) and education are taken into account as confounders
Analysis and data evaluation	
8	Positive if appropriate statistical model is used to evaluate data
9	Positive if effect size of variables was presented or p-value 0.05 was shown or can be calculated

### Data Extraction

The following details were extracted from the studies: first author, year of publication, country where the study was performed, data source used, period of study, characteristics of the population (age and gender), sample size, occupational group, study design, outcome definition (definition of retirement timing), and peer reviewed (yes/no).

### Data analyses

First, from the included articles, an overview of all determinants that influence retirement timing and that were part of the article hypothesis was created. For example, if the aim of the paper was to test the relationship between work and health characteristics and retirement, then only work and health variables were included in the review. Confounders (e.g., age, gender) added to this study were not included in the data extraction as we focused only on determinants that were part of the hypotheses under study in any included article. Only results from multivariate analyses were used. Determinants were classified into relevant domains by three researchers (MS, RS & CB).

A sensitivity analysis was conducted including determinants from the selected articles, which were not covered in the hypotheses, e.g., because they were included in the analyses as confounders. In addition, we extracted determinants from studies that were excluded in the last step of the inclusion procedure because they did not contain hypotheses about retirement timing. This sensitivity analysis enabled comparison between determinants that were purposively investigated for their role in retirement timing and variables that were included in statistical models for retirement timing without a predefined hypothesis about their role.

## Results

### Study selection

**Fig. 1:**
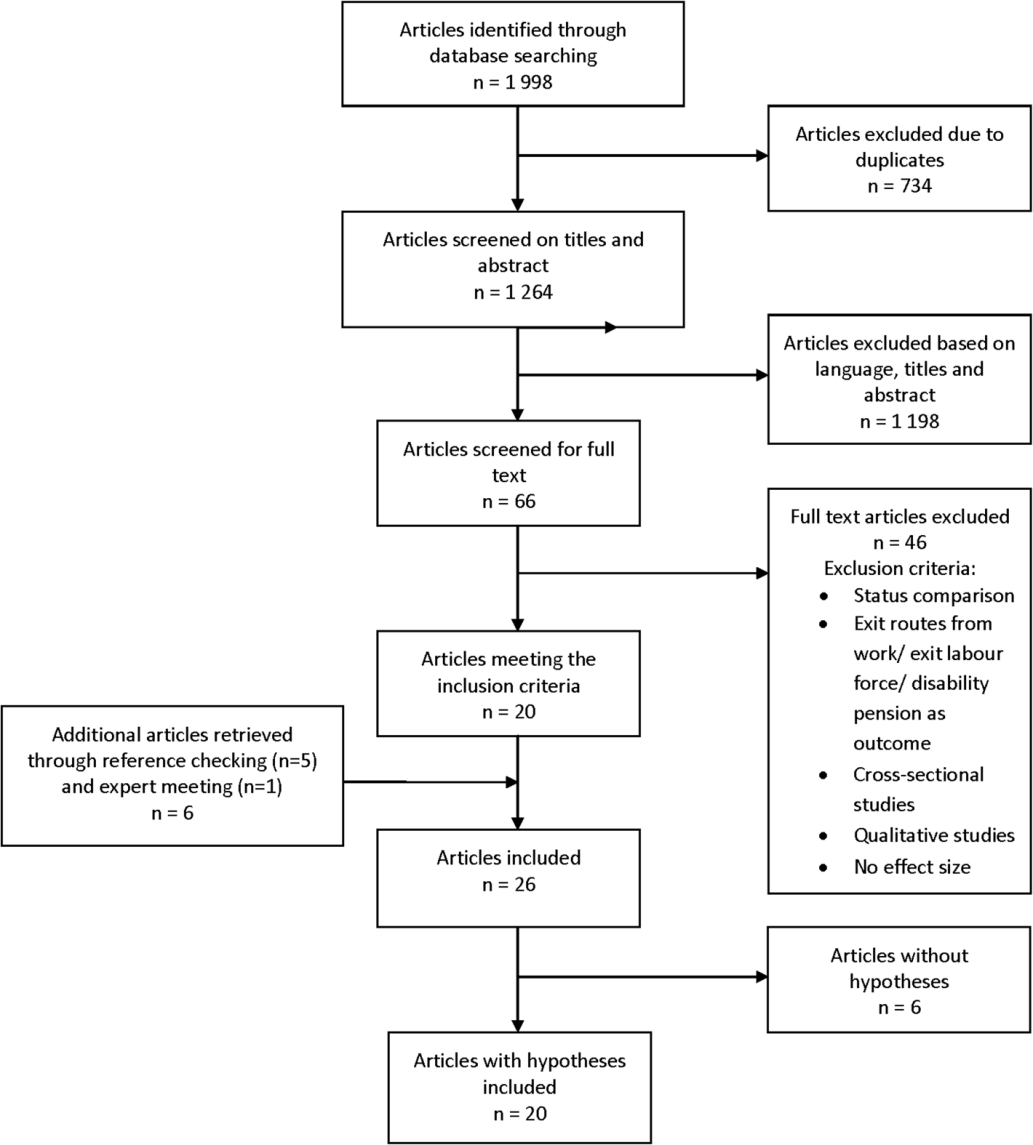
Flow diagram

The search strategy resulted in 1998 hits. After screening for duplicates, 1264 articles were screened by title and abstract. In total 1198 articles were excluded, because the articles were not written in Dutch or English, or were conducted among non-humans, the main outcome was other than time until retirement or the study had no longitudinal design. A total of 66 full texts were selected for further investigation. Finally, 20 articles met the inclusion criteria. Further reference checking resulted in five additional articles and the expert meeting yielded one additional article, resulting in a total of 26 articles. These articles were checked to determine if they reported one or more hypotheses. Ultimately, 20 articles reported hypotheses. Figure 1 presents the flow diagram.

### Methodological quality assessment

The outcome of the quality assessment is presented in Table 2. All articles, 20 in total, were considered to be of high quality.

**Table 2:** Results of the methodological quality assessment (+=positive; -=negative)

Study	Methodological quality									Total score	Total %	Quality
	1	2	3	4	5	6	7	8	9			
Christensen 2012 [24]	+	+	+	-	+	+	+	+	+	8/9	89	High
Coile 2000 [35]	+	+	+	-	-	-	+	+	+	6/9	67	High
de Preter 2013 [20]	+	+	+	+	-	+	+	+	+	8/9	89	High
Gesthuizen 2011 [22]	+	+	+	+	-	+	+	+	+	8/9	89	High
Gortz 2012 [25]	+	+	+	+	+	+	+	+	+	9/9	100	High
Herquelot 2011 [26]	+	+	+	-	-	-	+	+	+	6/9	67	High
Heyma 2004 [27]	+	+	+	-	-	+	-	+	+	6/9	67	High
Kerkhofs 1999 [28]	+	+	+	-	-	+	+	+	+	7/9	78	High
Marton 2010 [36]	+	+	+	-	-	+	+	+	+	7/9	78	High
Montizaan 2013 [33]	+	+	+	-	-	+	+	+	+	7/9	78	High
Olesen 2012 [29]	+	+	+	-	-	+	-	+	+	6/9	67	High
Ӧrestig 2013 [37]	+	+	+	-	+	+	+	+	+	8/9	89	High
Roberts 2009 [30]	+	+	+	+	-	+	+	+	+	8/9	89	High
Robroek 2013 [31]	+	+	+	-	-	+	+	+	+	7/9	78	High
Rubb 2009 [23]	+	+	+	-	-	+	+	+	+	8/9	89	High
Schils 2008 [21]	+	+	+	+	-	+	+	+	+	7/9	78	High
Schuring 2013 [32]	+	+	+	-	+	+	+	+	+	8/9	89	High
Song 2008 [12]	+	+	+	-	+	-	-	+	+	6/9	67	High
van Solinge 2010 [7]	+	+	+	+	-	+	+	+	+	8/9	89	High
van Solinge 2011 [34]	+	+	+	-	-	+	-	+	+	6/9	67	High

### Study characteristics

Significant variation existed among the included articles (see Table 3). Most studies were performed in European countries (e.g., Denmark, the Netherlands, France, Sweden, United Kingdom, and Germany); some studies were performed in Australia or the United States. The majority of the articles used data sources from the early 1990s until 2009. From the 20 articles, six articles reported separate analyses for men and women. Another three articles included only men in the analyses, while one article included only women. Four articles focused on a specific occupational group, such as day care teachers. Moreover, 13 articles used self-reported retirement as the main outcome, while six studies used register-based retirement as the main outcome. One study used a combination of register-based and self-reported outcomes.

**Table 3:** Characteristics of the studies

First Author + year of publication	Country + Dataset	Period of study	Characteristics population + Sample size	Occupational group	Outcome source	Peer-reviewed
Christensen 2012 [24]	Denmark, general population random sample	1985-2001	Workers and unemployed persons looking for a job in 1985 aged 50 (*N*= 9329)	Various	Register-based	Y
Coile 2000 [35]	United States, HRS	1951-1998	Male workers aged 55-69 (*N*= 5886)	Various	1) Before 1992 register-based, 2) From 1992 self-reported	Y
de Preter 2013 [20]	Europe, ECHP	1994-2001	Workers aged 50+ years (*N*= 3081 males, *N*=1413 females)	Various	Self-reported	Y
Gesthuizen 2011 [22]	The Netherlands, Dutch Socio-economic panel	1990-2001	Workers aged 50-65 (*N*=1521 males, *N*=1808 females)	Various	Self-Reported	N
Gortz 2012 [25]	Denmark, Longitudinal Data Set	1997-2006	Female workers aged 60-64 (*N*= 4800)	Day-care teachers and assistants	Register-based	Y
Herquelot 2011 [26]	France, GAZEL	1989-2007	Male workers aged 40-50, female workers aged 35-50 (*N*= 3036)	Workers of the French national electricity and gas company	Self-reported	Y
Heyma 2004 [27]	The Netherlands, CERRA	1993, 1995	Workers aged 40-65 (*N*= 4727)	Various	Self-reported	Y
Kerkhofs 1999 [28]	The Netherlands, CERRA	1991-1995	Main income earners aged 43 - 63 (*N*= 2560)	Various	Self-reported	Y
Marton 2010 [36]	United States, HRS	1992-2004	Male workers aged 51–61 (*N*=3150)	Various	Self-reported	N
Montizaan 2013 [33]	United States, US NLSOM	1966-1983	Male workers aged 45-59 (*N*= 3624)	Various	Self-reported	Y
Olesen 2012 [29]	Australia, HILDA	2001-2006	Workers aged 45-75 (*N*= 1516 males and 1287 females)	Various	Self-reported	Y
Ӧrestig 2013 [37]	Sweden, PSAE	2003-2007	Persons aged 57-64 (*N*=854)	Various	Register-based	Y
Roberts 2009 [30]	United Kingdom and Germany, panel survey	1991-2002	Workers aged 50-60/65 (female/male) (Germany: *N*= 1186 UK: *N*=1135)	Various	Self-reported	N
Robroek 2013 [31]	Sweden, Denmark, the Netherlands, Belgium, Germany, Austria, Switzerland, France, Italy, Spain, and Greece, SHARE	2004-2009	Workers aged between 50 and the statutory retirement age (*N*= 4923)	Various	Self-reported	Y
Rubb 2009 [23]	United States, supplements of the Current Population Surveys	1994-2001	Workers aged 55-64 (*N*= 5709 males and *N*=4917 females)	Various	Self-reported	Y
Schils 2008 [21]	United Kingdom, Germany, the Netherlands, panel survey	1991-2004, 1990-2005, 1990-2001	Workers aged 50-65 (Germany: 5150 The Netherlands: 1580 United Kingdom: 3629)	Various	Self-reported	Y
Schuring 2013 [32]	The Netherlands, POLS	1999-2008	Workers aged 45-64 (*N*= 3751)	Various	Register-based	Y
Song 2008 [12]	United States, Social Security administration data	1997-2005	Workers born in 1923-1943 (*N*= 124 850)	Various	Register-based	Y
van Solinge 2010 [7]	The Netherlands, NIDI	2001 and 2006/2007	Workers aged 50-60 (*N*= 1621)	Civil servants and employees active in ICT, retail, trade, industry and banking	Self-reported	Y
van Solinge 2011 [34]	The Netherlands, NIDI	2001 and 2006/2007	Workers aged 50-60 (*N*= 1611)	Civil servants and employees active in ICT, retail, trade, industry and banking	Self-reported	Y
Articles included in sensitivity analysis						
Coile 2007 [48]	United States, HRS	1951-2000	Male workers aged 55-69 (*N*=2467)	Various	1) Before 1992 register-based, 2) From 1992 self-reported	Y
de Preter 2013 [9]	Europe, SHARE	2004-2007	Workers aged 50-70 (*N*= 5127)	Various	Self-reported	Y
Friis 2007 [49]	Denmark, Database for Labor Market Research	1993-2002	Female workers aged 51-59 (*N*= 5538)	Nurses	Register-based	Y
Jensen 2012 [50]	Denmark, insurance fund PENSAM	1993-2008	Workers (*N*=2792)	Nurses aides	Register-based	Y
Mein 2000 [51]	United Kingdom, Whitehall II study	1988-1995	Workers aged 50 - 59.5 (*N*= 2532)	Civil servants	Self-reported	Y
Palmore 1982 [43]	United States, Ohio, NLS	1966-1976	Male workers aged 68-69 (*N*= 295)	Various	Self-reported	Y

Abbreviations: *CERRA* Centre for Economic Research on Retirement and Ageing, *ECHP* European Community Household Panel, *GAZEL* The GAZ and ELectricité cohort, *HILDA* Household Income and Labour Dynamics in Australia, *HRS* Health and Retirement Study, *NIDI* Panel study on retirement behaviour in the Netherlands, *NLS* The National Longitudinal Surveys, *POLS* Permanent Survey on Living Conditions, *PSAE* Panel Survey on Ageing and the Elderly, *SHARE* Survey of Health, Ageing and Retirement in Europe, *US NLSOM* US National Longitudinal Survey of Older Men

### Determinants of retirement timing

The determinants of retirement timing were categorized into the following eight domains: demographic factors (two determinants), health factors (12 determinants), social factors (one determinant), social participation (five determinants), work characteristics (21 determinants), financial factors (four determinants), retirement preferences (one determinant), and macro effects (three determinants). An overview of these domains, including the corresponding articles, is presented in Table 4.

**Table 4:** Domains, including the number of determinants, studies and references

Domain	# of determinants	# of studies	References
Demographic factors	2	3	[21–23]
Health	12	12	[7, 20, 21, 24–32]
Social factors	1	1	[21]
Social participation	5	1	[20]
Work characteristics	21	8	[20–22, 25, 27, 31, 33, 34]
Financial factors	4	5	[20, 28, 32, 35, 36]
Retirement preferences	1	1	[37]
Macro effects	3	3	[12, 22, 28]

From Table 4 it can be seen that two studies reported four domains in their study, however the four domains differed in the two studies [20, 21]. The domains reported by the majority of the articles were health and work characteristics, whereas the domains of social factors, social participation and retirement preferences were reported by only one study. No study included all eights domains. The lowest number of included domains was one (among six studies).

From Table 5 it can be seen that three articles included *demographic factors* in their hypotheses [21–23]. Education and gender were determinants in this domain. Operational definitions of education varied considerably between the articles. Gesthuizen and Wolbers [22] concluded that in the Netherlands lower routine non-manual, and skilled and unskilled manual male workers were more likely to exit employment and thus retire early compared to other occupational classes. Another study found that overeducation had no effect on early retirement in the United States [23]. Schils [21] showed that a higher educational level increased the probability of early retirement for the United Kingdom, but education did not influence early retirement in the Netherlands and Germany. Furthermore, Schils [21] found that in the Netherlands, women were less likely to retire early, while in the United Kingdom, women were more likely to retire early.

**Table 5:** Overview of determinants of retirement timing according to countries from articles with hypotheses

	The Netherlands	Denmark	Sweden	Germany	France	UK	Europe	USA	Australia
Demographic factors									
Education (high vs low)	[21, 22]			[21]		[21]		[23]	
Gender (female)	[21]			[21]		[21]			
Health									
Having a disease (y/n)		[24]			[26]		[20]		[29]
# days of treatment		[24]							
# of admissions		[24]							
General health									
Poor health	[21, 27, 28, 32]	[26]		[21]		[21]	[31]		
Subjective life expectancy	[7]								
Health limitations				[30]		[30]			
Latent health				[30]		[30]			
Lifestyle									
Overweight; obese vs normal							[31]		
Physical activity (low vs high)							[31]		
(ex-)smoker vs non-smoker							[31]		
Excessive alcohol intake (y/n)							[31]		
Social factors									
Partner employed (y/n)	[21]			[21]		[21]			
Social participation									
Providing care (y/n)							[20]		
Member of a club (y/n)							[20]		
Following general or higher education (y/n)							[20]		
Following vocational or training course (y/n)							[20]		
Satisfaction with leisure time (y/n)							[20]		
Work characteristics									
Working fulltime	[22]						[20]		
Hourly wage	[21]			[21]		[21]			
Tenure before age of 50 years	[21]			[21]		[21]			
Sector of work	[22, 27]								
Occupational class (lower vs upper)	[22]								
Irregular work (y/n)	[34]								
Larger firm size	[22]								
Job demands									
Physically demanding job	[34]						[31]		
High time pressure	[34]						[31]		
Job satisfaction (low vs high)							[20]		
Low job control							[31]		
Low rewards							[31]		
Challenge at work	[34]								
Contextual factors									
Firm specific training								[33]	
Child to teacher ratio in day-care sector		[25]							
Training opportunities	[34]								
Place to work/ time flexibility	[34]								
Use of seniority scheme	[34]								
Opportunities to grow	[34]								
Retirement behaviour among colleagues	[34]								
Support supervisor prolonged work participation	[34]								
Financial factors									
Higher personal income							[20]		
Social security wealth								[35]	
*Health insurance coverage* Employer provided and RHB; non-employer; none vs employer provided but no RHB								[36]	
Replacement rate (% of income a worker receives when ER, DP, unemployed)	[28]								
Retirement factors									
Retirement preferences: earlier vs later			[37]						
Macro effects									
Policy change (RET/FRA) (y/n)								[12]	
Birth cohort (related to pension regime) (≥1946=reference)									
≤1939	[22]								
1940-45	[22]								
Calendar time effects	[28]								

Abbreviations: *DP* disability pension, *ER* early retirement, *FRA* full retirement age, *RET* retirement earnings test, *RHB* retiree health benefits

For the domain *health*, 12 determinants were reported in the hypotheses of 12 articles [7, 20, 21, 24–32]. In addition, two sub-domains were found: general health and lifestyle. The most often studied determinant in the sub-domain general health was poor health. One study showed that poor health influenced early retirement in the United Kingdom and Germany, but it did not influence early retirement in the Netherlands [21]. In contrary, three studies conducted in the Netherlands [27, 28, 32] concluded that those with poor health were more likely to retire early.

A study conducted in France showed that people with diabetes were more likely to retire early compared to people without diabetes [26]. Christensen [24] found that having a chronic disease (mental or behavioural disorders, and nervous, respiratory, musculoskeletal, endocrine, nutritional, metabolic diseases) was associated with retirement timing in Denmark. Besides, more hospital admissions and more days of treatment were determinants of retirement timing as well [24].

Regarding gender differences in the effect of poor health on retirement, a study conducted in Australia, found that men with poor mental health were more likely to retire early [29], while no effect was found for women. Likewise, De Preter et al [20] found that mental health problems were associated with retirement timing among men, while this was not the case for women. A study conducted among female Danish day-care teachers showed no effect of poor health on early retirement after adjusting for other variables [25].

Roberts [30] showed that among British men, having experience of health limitations was associated with retirement timing, while this was not the case among British women. This study also showed that in Germany, fair health, good health and excellent health were predictors of retiring earlier among men. Among women, this relation was found for fair, good, and latent health [30].

The sub-domain lifestyle was reported by only one study [31]. They showed that lifestyle factors such as BMI, smoking and physical activity did not influence early retirement in Europe, except for excessive alcohol intake [31]. Furthermore, another study conducted in the Netherlands found that subjective life expectancy did not influence retirement timing [7].

In the domain *social factors*, one article studied the determinant partner employed in three countries [21]. This study showed that workers with an employed partner were less likely to retire early in Germany and the United Kingdom, while in the Netherlands no effect was found [21].

The domain *social participation* included five determinants reported in one article [20]. The majority of the determinants differed for men and women. This study found that in Europe, men who were member of a club, provided care or were satisfied with their leisure time were more likely to retire later, while both men and women who had followed an educational or vocational course were more likely to retire earlier. Women, on the other hand, were only more likely to retire later if they were satisfied with their leisure time [20].

Eight articles reported 21 determinants in the domain *work characteristics* [20–22, 25, 27, 31, 33, 34]. A study conducted in the Netherlands concluded that factors, such as temporary employment and working in small firms were associated with retiring early among men, and among women working in small firms was associated with retiring later [22]. De Preter et al [20] concluded that working full time was not associated with retirement timing for both men and women in Europe. One study concluded that working in industries or the public sector did not affect retirement timing, while working in the construction or catering sector was associated with retire earlier in the Netherlands [27]. Furthermore, Schils [21] showed that tenure before the age of 50 resulted in early retirement in the United Kingdom and Germany.

The other determinants were organized into two sub-domains, including job demands and contextual factors. Regarding job demands, having high time pressure or a physically demanding job did not influence retirement decisions in the Netherlands or Europe [31, 34]. In addition, having challenge at work was associated with retiring later in Europe[34]. In addition, low job control was a predictor for early retirement in the Netherlands [31].

Regarding contextual factors, one study showed that the child to teacher ratio in the day care sector did not have an effect on retirement timing in Denmark [25]. Receiving firm specific training was associated with retiring later among men in the Netherlands [33]. However, having training opportunity, time flexibility, use of seniority scheme, opportunities to grow, and retirement behaviour among colleagues did not influence retirement timing in the Netherlands [34]. However, this study showed that Dutch workers retire later if their supervisor supports prolonged work participation [34].

The domain *financial factors* included four determinants reported by five articles [20, 28, 32, 35, 36]. From these articles, three articles included personal income as a determinant in their hypotheses.

De Preter et al. [20] showed that in Europe, having a higher income was associated with retiring later among women. Contrary, a study conducted in the Netherlands showed that personal income did not influence early retirement [32]. In addition, replacement rate was also not associated with retirement timing in the Netherlands [28]. In the United States, men were not influenced by the social security wealth to retire [35] and also not by health insurance coverage employer provided and retiree health benefits, non-employer health insurance coverage or none [36].

One study included various retirement preferences in the domain *retirement preferences,* which was summarized into one determinant [37]. A Swedish study concluded that retirement preferences did not play a role in retirement timing [37].

Three studies included determinants about *macro effects* in their hypotheses [12, 22, 28]. Policy reforms, and birth cohort (i.e. used to investigate changes over time) are examples of these determinants. Although Gesthuizen and Wolbers [22] were unable to study the full scope of macro effects in the Netherlands (1990-2001), and they concluded that cohorts did not differ in their risk in late career instability. Another study conducted in the Netherlands, showed that calendar time effects did not influence early retirement [28]. A study conducted in the United States showed that changes in policy, such as the removal of the retirement earnings test and the increase in the full retirement age, may have affected retirement timing.

### Sensitivity analysis

Table 3 presents the characteristics of the six studies that were added to the sensitivity analysis. The sensitivity analysis showed that when including determinants that were not part of hypotheses related to retirement timing, the same domains remained.

Except for the increase in number of determinants, no major differences were found regarding the domains of *social participation, work characteristics,* and *retirement preferences*. Regarding the domain of *demographic factors*, the determinants of age, race and living in a city were included in the analysis of the articles without hypotheses. The determinant age was distinctly operationalized among the articles, e.g., age was taken into account as a continuous variable, categorical variable or dichotomized. Furthermore, another determinant emerged in the domain *health,* namely sick leave. In the domain *social factors*, determinants covering family factors (e.g., information on spouse, children, grandchildren, parents) were identified. With regard to the domain *financial factors*, determinants on mortgage and possessions were also included when considering articles without hypotheses. Moreover, in the domain *macro effects*, the effect of countries on retirement timing was included in the list of determinants.

## Discussion

In this narrative systematic literature review we explored the determinants of retirement timing among older workers from both an economic and occupational health perspective. Twenty articles reported determinants of retirement timing in modern industrialized countries, which resulted in 49 determinants of retirement timing. All determinants were classified into eight domains: demographic factors, health, social factors, social participation, work characteristics, financial factors, retirement preferences, and macro effects.

A previous systematic review on retirement timing investigated the antecedents, moderators and consequences of early, on-time, or later retirement [38]. This review is in line with the present review since not only health and work characteristics influenced the retirement process, but family factors, economic status, and macro effects as well. Furthermore, the domains presented in research frameworks of lidA and STREAM correspond to the domains found in the current review that are involved in the decision to retire (early). This may suggest that these frameworks represent an overview of domains from various disciplines (e.g., occupational health and economics). Therefore, it is recommended to apply a multidisciplinary approach in future research on retirement timing.

Another way to look at determinants of retirement timing is by distinguishing push and pull factors. Push factors relate to negative considerations that lead to retirement, and comprise the domains health, work characteristics, demographics and macro effects. Pull factors make retirement more attractive than being employed. Examples of pull factors are found in the domains retirement preferences, social factors, social participation and financial factors. In total, 18 different studies included push factors, while only 6 different studies included pull factors. Therefore, future research should also include pull factors that influence retirement timing in order to gain a clearer picture on how push and pull factors interact with retirement timing.

The focus on health and work in retirement research has been illustrated by previous systematic reviews on exit from paid employment (i.e. disability pension, unemployment and early retirement), and they focused mainly on the push domains health and work characteristics [39–41]. In addition, unlike these reviews that include retirement status as an outcome, the present study included only studies with the outcome time until retirement. The timing of retirement might provide additional information on determinants that is lost when retirement status is investigated at a specific point in time, thus comparing groups of workers who have or have not retired without taking into account their exact retirement dates.

Surprisingly, having a physically demanding job and high time pressure in the domain work characteristics did not show an association with retirement timing. This might be explained by the healthy worker effect [42]. Workers who are not able to perform in a physically demanding job or having high time pressure at work may have changed jobs or may have left the workforce earlier, leaving a selection of relative healthy older workers.

It might be interesting to make a distinction between countries that have mandatory retirement (i.e., the employment contract of employees stop when they reach the statutory retirement age) and countries that do not have mandatory retirement (i.e., employment contracts do not stop at a certain age). A study conducted in the United States found that men subjected to mandatory retirement retired later [43]. Since only one study investigated the influence of mandatory retirement on retirement timing, it is not possible to draw conclusions. Future research should investigate this further, especially since it is likely that more countries will abolish mandatory retirement in the near future. In addition to investigating the difference in retirement timing for countries with and without a mandatory retirement, it would also be interesting to investigate the influence of the abolishment of mandatory retirement within a country on retirement timing.

Besides, other differences among countries are likely since pension benefits, social security- and retirement schemes differ between countries. For example, in the United Kingdom and Germany people retired at specific ages (i.e. 62 years) before the official retirement age, while in the Netherlands people retired between 60 and 62 years old. At the same time, disability and unemployment benefits were more generous in the Netherlands and Germany than in the United Kingdom, leading to more different exit routes from paid employment [21]. Moreover, before the Patient Protection and Affordable Care Act, also known as Obamacare, citizens of the United States were not obliged to have an health insurance. Therefore, people with a chronic disease were probably more likely to work until the official retirement age to claim an health insurance supported by their employer.

Another difference between the United States and most of the European countries may be the more generally accepted use of private pensions instead of employer pensions. Both pensions are defined contribution pensions, which means that the income received during retirement is based on the money that is paid in and invested in a retirement pension. The difference between these two pensions is that a private pension is set up by an individual him- or herself, while a workplace pension is set up by an employer. Employer pensions provide some benefits compared to private pensions. First, group saving is more cost-effective than individual saving and employer pensions can partly offset risks, such as outliving your pension income [44]. Second, employer pensions were likely to have substantial retirement incentives [45]. To illustrate, private pensions mean that employees are individually responsible for their private pension plans and thereby for their economic security in the future [46]. This may result in working longer to reach a sufficient amount of income to maintain pre-retirement standard of living in retirement. These examples imply that country specific context is important in discussing retirement timing, but also that determinants on macro/country level are not well investigated yet.

A strength of this systematic review is that we used multiple databases to combine knowledge from both occupational health and economics. Furthermore, the results are based on determinants that were part of one or more hypotheses in the articles to prevent finding inconclusive results due to chance findings. However, there were also limitations in this study. First, we included articles using self-reported retirement as well as register-based retirement. A limitation of self-reported retirement is that persons with more than one employment status may have reported their status differently. On the other hand, register-based information relies on income categories, which may differ from how people evaluate their own situation. However, it should be kept in mind that determinants may relate uniquely to self-reported retirement compared to register-based retirement. This may at least partially explain the disparate findings per (domain of) determinant(s). Second, we also included non-peer-reviewed articles. The reason for this is that in economics, many articles are published as working papers and therefore are not (yet) peer-reviewed, while they are important for the field of economics. Third, the results are based on data from multiple modern industrialized countries. Studies on retirement timing in non-modern industrialized countries are missing in this narrative review as these countries do not have a pension system. Worldwide approximately only 20 percent of the population receives social security [47]. Fourth, it is difficult to conduct meta-analyses on the effects of the determinants since there are various ways of how determinants are operationalized. In addition, since this systematic review was exploratory and included articles from multiple disciplines, a wide variety of statistical models and frameworks were used. Therefore, it is difficult to compare the effect sizes of the determinants. Finally, due to the recent policy changes, the determinants that affect retirement timing may change in the future.

### Implications for research

Future studies on retirement timing should include both men and women in the population as the composition of the current labour market has changed. However, further research is also needed to investigate whether these determinants are unique for men and women, implying that analyses should be performed separately as well. There is also a need for the availability of data that includes determinants from all domains. In this way, it becomes easier for researchers to study multiple domains when studying retirement timing.

In addition, more research is needed to study the effect of ongoing changes in the labour market, such as the increase of self-employment. Also the role of private pensions in retirement timing may be an interesting direction for future research as it is becoming a more relevant source of income after retirement in many countries and therefore might influence retirement timing. More research is needed on how all domains interact, because each domain or determinant may push or pull in a distinct direction in retirement timing (e.g., financial incentives versus poor health). Finally, more research is needed to study the effects of policies, such as the availability of mandatory retirement or disability pensions, on retirement timing should become more common.

## Conclusion

It is important to consider multiple disciplines when answering research questions about retirement timing. This literature review showed that there are 49 determinants of retirement timing for modern industrialized countries, which can be summarized into eight domains: demographic factors, health, social factors, social participation, work characteristics, financial factors, retirement preferences, and macro effects. Finally, this narrative literature review also showed that retirement timing is not equally studied around the world.

## Additional file

Additional file 1: Search terms Web of Science. This table gives the search terms used for Web of Science**.** (PDF 138 kb)

## Abbreviations

CERRA: Centre for Economic Research on Retirement and Ageing
DP: Disability pension
ECHP: European Community Household Panel
ER: Early retirement
FRA: Full retirement age
GAZEL: The GAZ and ELectricité cohort
HILDA: Household Income and Labour Dynamics in Australia
HRS: Health and Retirement Study
LidA: Leben in der Arbeit
NIDI: Panel study on retirement behaviour in the Netherlands
NLS: The National Longitudinal Surveys
POLS: Permanent Survey on Living Conditions
PSAE: Panel Survey on Ageing and the Elderly
RET: Retirement earnings test
RHB: Retiree health benefits
SHARE: Survey of Health, Ageing and Retirement in Europe
STREAM: Study on Transitions in Employment, Ability and Motivation
US NLSOM: US National Longitudinal Survey of Older Men
