# Supplementary material for: Domains and determinants of retirement timing: A systematic review of longitudinal studies
Source: BMC Public Health. 2018 Aug 31;18:1083. doi: 10.1186/s12889-018-5983-7 (PMC6119306; doi:10.1186/s12889-018-5983-7)
Supplement: Supplementary file 1 — Search terms Web of Science. This table gives the search terms used for Web of Science. (PDF 138 kb) [file 12889_2018_5983_MOESM1_ESM.pdf]

ADDITIONAL FILE 1

Search terms Web of Science

**#**	***Amount of articles***	***Search term***
#3	*451*	*#2 AND #1*<br><br>*Indexes=SCI-EXPANDED, SSCI, A&HCI Timespan=All years*
#2	*1.080.037*	*TS=("cohort" OR "longitudinal" OR "prospective" OR "retrospective")*<br><br>*Indexes=SCI-EXPANDED, SSCI, A&HCI Timespan=All years*
#1	*2.639*	*TS=("retir*" NEAR/2 ("time" OR "timing" OR "early" OR "earlier" OR "anticipat*" OR "prepar*" OR "transition*" OR "intention*" OR "expectation*" OR "application*"))*<br><br>*Indexes=SCI-EXPANDED, SSCI, A&HCI Timespan=All years*
